# Incidence, treatment and outcome of abdominal metastases in extremity soft tissue sarcoma: Results from a multi‐centre study

**DOI:** 10.1002/jso.25856

**Published:** 2020-01-31

**Authors:** Maria A. Smolle, Angelika Schaffler, Andreas Leithner, Veroniek M. Van Praag, Marko Bergovec, Joanna Szkandera, Bernadette Liegl‐Atzwanger, Maya Niethard, Per‐Ulf Tunn, Michiel Van De Sande, Dimosthenis Andreou

**Affiliations:** ^1^ Department of Orthopaedics and Trauma Medical University of Graz Graz Austria; ^2^ University Hospital Zurich Zurich Switzerland; ^3^ Department of Orthopaedics Leiden University Medical Centre ZA Leiden The Netherlands; ^4^ Division of Clinical Oncology, Internal Medicine Medical University of Graz Graz Austria; ^5^ Diagnostic and Research Institute of Pathology Medical University of Graz Graz Austria; ^6^ Department of Orthopaedic Oncology HELIOS‐Klinikum Berlin‐Buch Berlin Germany; ^7^ Tumour Orthopaedics and Sarcoma Surgery HELIOS Klinikum Bad Saarow Bad Saarow Germany

**Keywords:** abdominal metastasis, soft tissue sarcoma, survival

## Abstract

**Background and Objectives:**

Abdominal metastases (AM) from soft tissue sarcoma (STS) are rare and prognosis is poor. The aims of the study were to (a) identify risk factors for the development of AM and to (b) investigate the outcome of AM‐patients.

**Methods:**

Seven‐hundred‐sixty‐nine STS‐patients with localised disease at diagnosis treated at three tumour centres (2000‐2016) were retrospectively included (409 males; mean age, 55.6 years [range, 8‐96 years]; median follow‐up, 4.1 years [interquartile‐range, 2.5‐6.6 years]).

**Results:**

Two‐hundred‐two patients (26.3%) developed secondary metastases, and 24 of them AM (3.1%). Ten patients developed first AM (FAM) after a mean of 2.4 years and 14 patients late AM (LAM, after being diagnosed with metastases to other sites) after a mean of 2.0 years. Patients with liposarcoma had a significantly higher risk of developing AM (*P* = .007), irrespective of grading. There was no difference in post‐metastasis‐survival (PMS) between patients with AM at any time point and those with metastases to other sites (*P* = .585). Patients with LAM or FAM showed no difference in post‐abdominal‐metastasis‐survival (*P* = .884).

**Conclusions:**

Survival in patients with AM is poor, irrespective of whether they develop secondarily to other metastases or not. Patients at high‐risk of AM (ie, liposarcoma) may be followed‐up regularly by abdominal‐ultrasound/CT.

## 1 INTRODUCTION

Soft tissue sarcomas of the extremity and trunk (STS) are rare tumours with an estimated incidence of 2.4 cases per 100 000 persons per year.^1^ About 15% of STS‐patients develop local recurrences and 30% distant metastases at 5 years, most commonly to the lungs.^2^, ^3^, ^4^, ^5^ Other metastatic sites are described (bone and lymph nodes), however abdominal metastases (AM) from STS, are very uncommon.^6^, ^7^ AM carry a poor prognosis with 2‐year survival rates of 43%.^7^ Diagnosis can be difficult as AM may be asymptomatic for a long time or may only cause vague discomfort.^8^ Symptoms involve intestinal obstruction, abdominal pain, or gastrointestinal bleeding.^8^ Since these very symptoms can also represent side effects from chemotherapy (CTX) or pain management, they can be easily misinterpreted.^6^, ^8^ There is no clear consensus how to follow‐up patients with localised STS following surgical resection, with different studies describing different follow‐up regimes.^9^, ^10^, ^11^ The current ESMO guidelines^12^ suggest that STS‐patients should be followed‐up every 3 to 4 months for the first 3 years, then bi‐annually for the following 2 years and thereafter annually by chest‐X‐rays and/or computed tomography‐scan (CT‐scan) of the lungs. However, the impact of regular abdominal CT‐scans in follow‐up remains unclear.^12^

Three studies have been published so far on the topic of AM of STS.^7^, ^13^, ^14^ However, the number of patients with AM identified in these studies was relatively low and more importantly they did not distinguish between patients with AM as the first metastatic manifestation and those with AM developing after other metastases had occurred, therefore leaving many clinical questions unanswered.

Incidence of AM, risk factors for their development, diagnostic tools and impact on patient survival were analysed in the present retrospective multi‐centre study. The aims of the study were to (a) to investigate the outcome of these patients in comparison to STS‐patients with metastases to other sites and those without metastases and (b) to identify factors associated with a higher risk for developing AM.

## 2 MATERIALS AND METHODS

We performed a retrospective analysis of the files of 769 patients who had been diagnosed with a primary localised extremity STS (G1‐G3) between January 2000 and May 2016 at three tertiary tumour centres. Four‐hundred‐nine patients were male (53.2%) and 360 were female (46.8%). The mean age of all patients was 55.6 years (range, 8‐96 years). Median follow‐up was 4.1 years (IQR, 2.5‐6.6 years).

Data was collected by reviewing medical records, such as pathology and radiology reports, outpatient records and medical charts. Time to secondary metastasis (SM) and first abdominal metastasis (FAM) was calculated from date of definite surgery to the first radiological verification of a metastatic focus (eg, MRI, CT‐scan). Late abdominal metastasis (LAM) was defined as an abdominal metastasis developing after SM at another site. Follow‐up intervals were calculated from the date of primary surgery to the date of last follow‐up or death. Routine follow‐up was performed according to the ESMO^12^ and NCCN^15^ guidelines in the respective years, including alternate CT‐scans of the abdomen and abdominal ultrasonography, depending on the at each time valid policy.

Date of SM, as well as FAM and LAM, was documented. Overall‐survival (OS) was calculated from date of primary surgery to date of last follow‐up or death. Post‐metastasis‐survival (PMS) was defined as the interval between development of SM and last‐follow‐up or death. Post‐abdominal‐metastasis‐survival (PAMS) was calculated from date of development of AM to last follow‐up or death. The current study has been approved by the local institutional review board (EK‐Nr. 24‐573 ex 11/12) and has been performed in accordance with the Declaration of Helsinki.

### 2.1 Statistical analysis

All statistical analyses were performed with the *Stata* software version *15.1* (StataCorp, TX). Means and medians were calculated for normally and non‐normally distributed data using *t*‐tests and Mann‐Whitney‐U‐tests, respectively. Comparisons between groups were made using *χ* ^2^ tests. Kaplan‐Meier estimates and Cox‐regression models were used to estimate outcome variables, providing hazard ratios (HRs), 95% confidence intervals (95%CI) and *P* values. Considering that only two patients with AM underwent additional surgery, subgroup analysis to assess the effect of metastasectomy on PMS in this group of patients was not performed. Furthermore, the multivariate Cox‐regression analysis was limited to two factors, in accordance with the 'one in ten rule'.^16^ All *P* values are two‐sided; a *P* value of <0.05 was considered statistically significant.

## 3 RESULTS

Two‐hundred‐two patients (26.3%) developed SM after a median of 15 months (IQR, 10‐29 months). Demographic features and tumour as well as treatment specific details of patients with and without SM are depicted in Table 1.

**Table 1:** Comparison of patient‐ and primary tumour characteristics with secondary metastases and those without

N = 769	No SM	SM	Missing	*P* value
Mean age	55.9 y	54.9 y	0	.230
Sex				
Female	278	85	0	.116
Male	292	117		
Histology (primary tumour)				
Liposarcoma	165	31	0	**<.001**
Myxofibrosarcoma	101	31		
Leiomyosarcoma	55	28		
Synovial sarcoma	39	9		
UPS	98	23		
Other	109	80		
Grading (primary tumour)				
G1	187	22	15	**<.001**
G2	194	79		
G3	173	99		
Location				
Upper limb	107	45	0	.552
Lower limb	410	142		
Trunk	47	13		
Head/Neck	3	2		
Proximity				
Proximal	362	124	2	.588
Distal	181	64		
Median	24	12		
Depth				
Superficial	57	22	23	.826
Deep	489	178		
Tumour size	8.2 cm	8.8 cm	30	.215
Neoadj CTX				
No	523	175	0	**.018**
Yes	44	27		
Adj CTX				
No	525	177	0	**.032**
Yes	42	25		
Neoadj. RTX				
No	530	176	0	**.005**
Yes	37	26		
Adj RTX				
No	344	90	0	**<.001**
Yes	223	112		

Abbreviations: CTX, chemotherapy; RTX, radiotherapy; SM, secondary metastasis.

*P*‐values in bold indicate significant results.

**Figure 1:**
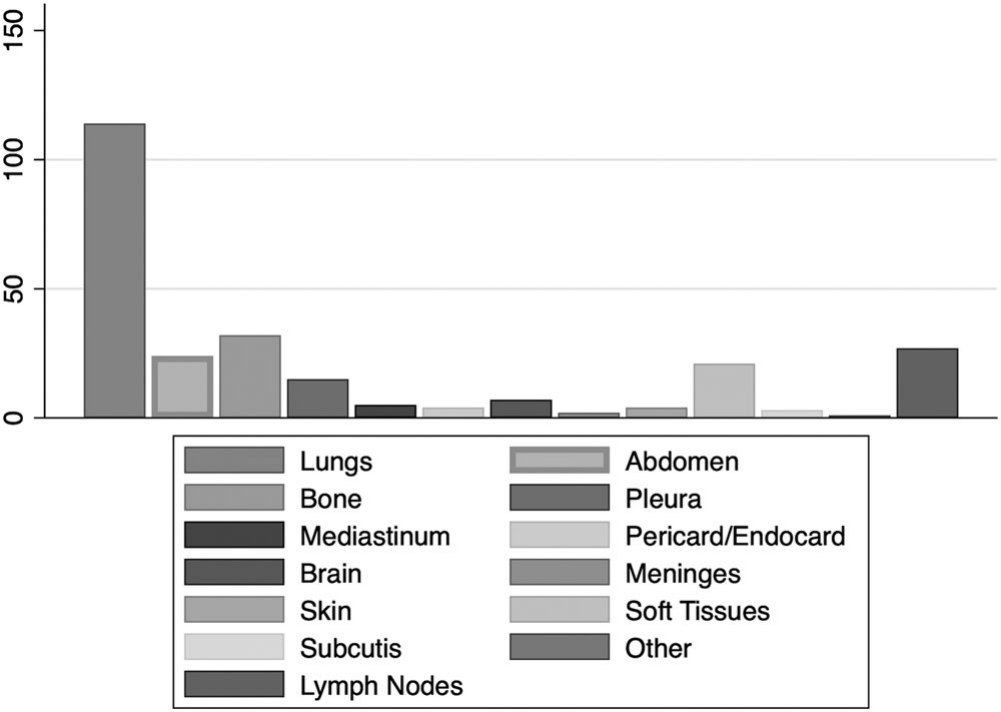
Frequency of first metastases to different body regions

Taking into account all metastatic foci developing during the course of the disease, the most common location was the lung (n = 114), followed by bone (n = 32) and regional as well as distant lymph nodes (n = 27; Figure 1). Rare locations included the pericard/endocard as well as the skin in four cases, the subcutis in three and the meninges in two cases.

Twenty‐four patients developed AMs during the course of disease (3.1%), including 13 hepatic, three intestinal, two pancreatic and two peritoneal metastases. Further four patients had multiple intestinal metastases, of whom two also had metastatic foci in the retroperitoneum. Ten patients presented with a FAM after a mean of 2.4 years (range, 7 months to 8.3 years) and 14 patients a LAM, after having developed a primary metastasis to another site. In the latter case, the meantime to LAM from the development of SM was 2.0 years (range, 1 month to 3.6 years). Moreover, hepatic FAM (n = 5) showed no tendency to develop earlier than FAM at other sites (n = 5; 17.1 vs 10.1 months; *t*‐test *P* = .793).

### 3.1 Diagnostic pathway

Nonabdominal SM was detected by CT‐scan of the thorax in 76 cases, followed by chest‐X‐ray in 20 cases, MRI in eight cases, ultrasound in one case and other methods (abdominal CT‐scan, PET‐CT) in 10 patients. In nine patients, the detection method of metastasis was unclear. Most AM, irrespective of their type, were detected by CT‐scan of the abdomen (n = 9) and ultrasound (n = 3).

The most common symptom reported by patients at diagnosis with AM included unspecific abdominal pain in one‐fifth of patients (n = 5).

### 3.2 Treatment

Primary treatment of AM included CTX in 18 patients, CTX+surgery in two patients (one for a hepatic metastasis, one for an acute ileus), CTX combined with embolization in one case and best supportive care in three patients.

There was no difference in the time interval until the development of FAM vs SM to other sites (mean time, 29.1 months vs 27.5 months; t‐test *P* = .875).

### 3.3 Risk factors for abdominal metastases

**Figure 2:**
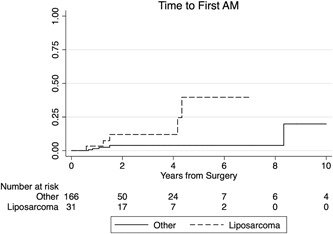
Risk of development of primary AM vs metastases to other sites from date of surgery. Patients with liposarcoma (dashed line) have a significantly higher risk of developing abdominal metastases (*P* = .016). AM, abdominal metastases

Of those 24 patients with AM, 11 had originally been diagnosed with liposarcoma as the underlying histological subtype, four with UPS, three with leiomyosarcoma, one with myxofibrosarcoma and five with a miscellaneous histology (*χ* ^2^ test; *P* = .178). Patients with liposarcomas developed FAM significantly more often than patients with other histologies (*P* = .016; Figure 2). Patients with myxoid liposarcoma had a significantly higher risk of developing FAM in comparison to the remaining histological subtypes pooled together (HR, 7.712; 95% CI, 1.920‐30.986; *P* = .004). On the other hand, there were no differences in the probability of developing AM between the different subtypes of liposarcoma (ie, NOS, pleomorphic and myxoid) in our patient cohort (Table 2).

**Table 2:** Univariate Cox‐regression model showing the risk for patients to develop initial AM vs SM from the date of surgery. Patients with liposarcoma have a significantly higher risk of developing AM

N = 197	Hazard ratio	Confidence interval		*P* value
		Lower	Upper	
Mean age	0.993	0.683	0.958	.683
Sex				
Female (ref)	1			.132
Male	0.536	0.238	1.206	
Histological subtype				
Others (ref)	1			**.016**
Liposarcoma	5.072	1.357	18.955	
Liposarcoma subtype				
Liposarcoma NOS (ref)	1			
Myxoid liposarcoma	3.111	0.862	11.220	.083
Pleomorphic liposarcoma	2.647	0.272	25.798	.402
Grading				
G1 (ref)	1			
G2	1.102	0.113	9.868	.931
G3	1.578	0.183	13.566	.678
Depth				
Superficial (ref)	1			.763
Deep	1.382	0.169	11.315	
Tumour size	1.027	0.913	1.156	.655
Adjuvant CTX				
No (ref)	1			.682
Yes	1.383	0.293	6.532	
Adjuvant RTX				
No (ref)	1			.746
Yes	0.815	0.236	2.818	

Abbreviations: AM, abdominal metastases; CTX, chemotherapy; NOS, not otherwise specified; RTX, radiotherapy.

*P*‐values in bold indicate significant results.

In the multivariate analysis, histological subtype only (liposarcoma vs others; *P* = .007) could be identified as an independent negative prognostic parameter regarding the development of AM as the first metastatic manifestation, irrespective of grading (Table 3).

**Table 3:** Multivariate Cox‐regression model showing the risk for patients to develop primary AM vs SM from date of surgery. In comparison to all other histological subtypes, patients with liposarcoma have a significantly higher risk of developing primary AM

N = 195	Hazard ratio	Confidence interval		
		Lower	Upper	*P* value
Histological subtype				
Others (ref)	1			**.007**
Liposarcoma	6.589	1.668	26.026	
Grading				
G1 (ref)	1			
G2	1.792	0.195	16.453	.606
G3	3.422	0.372	31.457	.277

*P*‐values in bold indicate significant results.

### 3.4 Outcome

At last follow‐up, 523 patients were alive without (68.0%) and 67 alive with disease (8.7%). One‐hundred‐nineteen patients had died of STS (15.5%), and 57 due to other causes (7.4%), whereas in four patients the cause of death remained unknown (0.4%). Depending on their metastasis status, nine patients with AM (37.5%), 86 patients with SM (51.1%) and 497 patients without metastases were still alive (87.8%).

**Figure 3:**
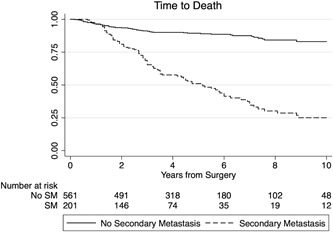
Survival curves for patients developing secondary metastasis (SM; dashed line) and those patients who did not (no SM; solid line; *P* < .0001)

**Figure 4:**
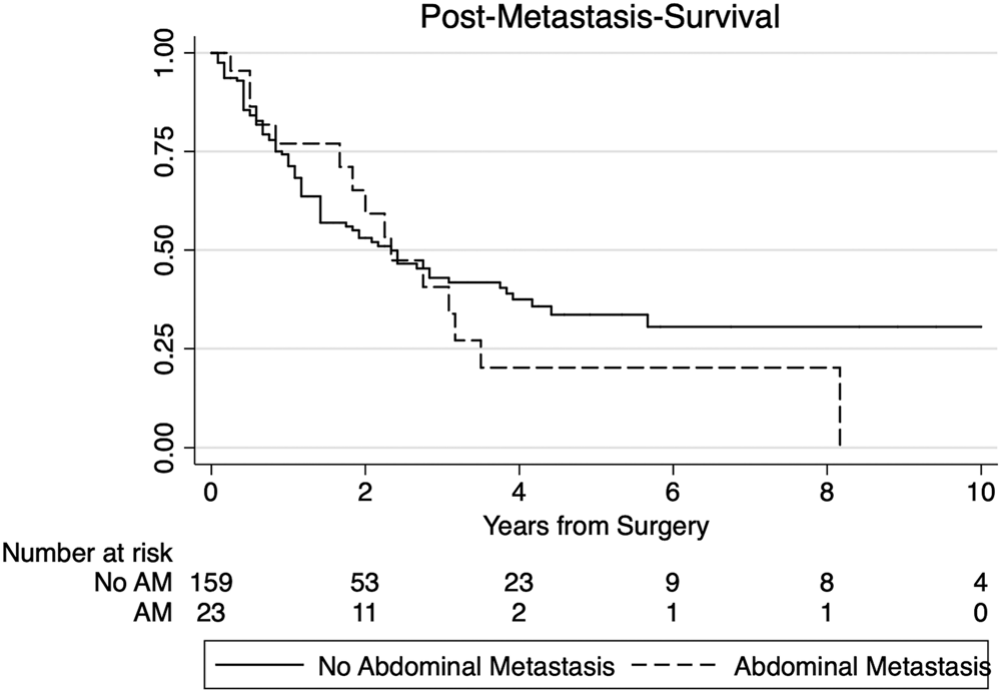
Difference in post‐metastasis survival between patients with SM other than abdominal metastasis (no AM; solid line) and those with AM (FAM+LAM, dashed line; *P* = .585), calculated from the date of onset of SM or AM. AM, abdominal metastases

Patients without SM had a significantly better OS than patients with SM (log‐rank *P* < .0001; Figure 3). There were no differences in PMS between patients with AM at any time point and patients with SM other than AM (*P* = .585; Figure 4). Patients undergoing surgery for their metastasis had a significantly better PMS than patients treated by CTX, radiotherapy (RTX) or best supportive care (HR, 0.544; 95%CI, 0.328‐0.902; *P* = .018).

Interestingly, there was also no difference in PAMS between patients with FAM and those with LAM (log‐rank *P* = .884), suggesting that occurrence of AM at any time point similarly reduces survival probability. Additionally, PAMS did not significantly differ between patients with liver metastases vs AM to other sites (HR, 1.610; 95%CI, 0.537‐8.833; *P* = .395).

## 4 DISCUSSION

STS metastasise most commonly via the bloodstream to the lungs.^17^ AM, on the other hand, are extremely uncommon.^8^ Few case reports and studies have been published, describing the course of patients with abdominal or retroperitoneal metastases from STS.^7^, ^13^, ^14^, ^18^, ^19^, ^20^ These studies either included relatively low numbers of patients with AM or did not describe factors associated with a higher risk of developing AM in a time‐dependent manner.

Thompson et al^21^ described 140 STS‐patients who were screened with CT‐scans of the abdomen, and found that a total of four patients (2.9%) developed AM.^21^ In another single‐centre study, 19 AMs developing during the course of disease were observed in a group of 2127 STS (<1%).^7^ In our cohort, 24 out of 769 patients with localised extremity and trunk STS developed AMs‐most commonly to the liver‐resulting in a total frequency of 3.1%. This discrepancy may be explained by the fact that in our collective, most patients with AM were diagnosed by CT‐scans (followed by ultrasonography), whilst in the study by Behranwala et al,^7^ the use of abdominal CT‐scans was not clearly described. Therefore, some AM may have remained undiagnosed in that study, explaining the lower rate.

Raising the question why AM from STS are extremely rare, one has to look at to basic research; according to two experimental studies by Skubitz et al,^22^, ^23^ STS have different metastatic propensities based on their gene expression patterns. This suggests that not only the histological subtype, tumour size and grading have an influence on the propensity of STS‐metastasis to occur, but also the individual genetic profile of STS most probably results in a different affinity to specific tissues.

Assuming that the affinity of STS to viscera is low, the time interval until development of AM should also be rather long. Indeed, in our cohort it took a mean of 2.4 years for FAM to occur, while the mean interval until development of LAM following first metastases to other sites was also long (2.0 years). This is comparable to the 2.3 years interval observed by Behranwala et al^7^ for patients with any AM. On the other hand, initial metastases to, for example, lungs and bones developed after a median of 15 months in our cohort.

In our study, patients with liposarcomas as the underlying histological subtype had a significantly higher risk of developing AM, irrespective of grading. This is corresponding to observations made by Behranwala et al^7^ and Lev‐Chelouche et al,^6^ in whose studies six out of 19 patients (myxoid subtype) and four out of 10 patients with AM, respectively, had liposarcomas as the underlying histotype.^6^, ^7^ In our collective, patients with liposarcoma NOS, a myxoid or pleomorphic subtype had an equally high risk of developing AM. Of note, myxoid liposarcomas tend to metastasise at higher rates to sites other than the lung (including bone and abdomen/retroperitoneum) in comparison to most STS subtypes.^7^, ^24^ This observation could be confirmed in the present study, with myxoid liposarcoma‐patients having significantly higher risks of developing AM as compared with all other histologies pooled together. However, we did not find a higher risk of AM for myxoid liposarcoma‐patients when compared to other liposarcoma subtypes, indicating that at least in the frequency of abdominal surveillance (ie, CT‐scans, sonography), no difference between the liposarcoma subtypes should be made.

The development of AMs at any time point was associated with a similar reduction in survival probability in our collective. This suggests that once STS gain affinity to the viscera, they have already converted into more aggressive tumours.

A recent study has shown that surgical resection of STS‐metastases, in general, may be associated with an improved survival outcome, irrespective of confounding factors.^25^ In that study, however, most metastases were located in the lungs and soft tissues rather than the abdomen. In our cohort, most patients with AM were administered CTX, whilst two patients underwent additional surgery. Consequently, we only analysed the effect of metastasectomy on PMS for all patients rather than patients with AM only, revealing that surgery for metastases was associated with an improved outcome.

Due to the retrospective design of the study, not all questions can be answered, though, including the issue whether an earlier detection of AM would result in a survival benefit and at which frequency to perform abdominal ultrasound or CT‐scans. These issues may be addressed in the frame of a prospective study, similar to the study by Puri et al,^26^ with patients assigned to various standardised protocols differing in intervals and methodology. Recently, two apps (Sarculator,^27^ PERSARC^28^) have been developed based on a thorough analysis of the risk of local recurrence and distant metastasis as well as OS in patients with eSTS, aiming at individualising patient treatment and aftercare. Based on these advancements, follow‐up may in the future slightly differ from the proposed ESMO guidelines,^12^ taking into account individual risk factors altering rates of LR and DM, as well as overall prognosis.

There are some major limitations to the present study. As three different centres participated and patients over a long time period where included, the surveillance‐schemes may have changed over the years, thus not guaranteeing that AM were detected as often and early at every period. Moreover, due to the retrospective design of this study, confounding factors as patient symptoms, leading to the conduction of an abdominal CT‐scan outside routine follow‐up, cannot be eliminated. Additionally, no data on diagnostic delay of AM or SM was uniformly available, wherefore no conclusion on whether earlier detection of AM would alter patient prognosis could be drawn. Furthermore, due to the low rate of AM in the present collective, the number of variables being analysed in the multivariate setting was limited. On the other hand, we were able to include a very large number of patients with eSTS, treated according to at the time current guidelines at experienced tertiary referral sarcoma centres, factors which we believe largely offset the impact of the above limitations.

## 5 CONCLUSIONS

AM from soft tissue sarcoma constitutes a very rare event. Survival is likewise reduced in patients with first AM and those with late AM, signifying that tumours developing abdominal metastasis are equally aggressive, and that outcome is generally poor. Patients with liposarcomas appear to be at a significantly higher risk of developing AM, wherefore at least in this these patients, surveillance with abdominal CT‐scans or sonography in follow‐up should be considered. Nevertheless, prospective, randomised studies are warranted to investigate the frequency and methodology of future follow‐up protocols.

## CONFLICT OF INTERESTS

Author MvdS reports grants from Daiichi Sankyo, outside the submitted work. Author AL reports grants from Johnson & Johnson and Alphamed, outside of the submitted work. The remaining authors have no conflicts of interest relating to the present manuscript to declare.

## ETHICS STATEMENT

The current study has been carried out in accordance with the ethical standards of the Declaration of Helsinki and has been approved by the local ethics committee (EK‐Nr. 24‐573 ex 11/12).

## SYNOPSIS

Herein, the incidence of abdominal metastases (AM) after curative extremity‐soft‐tissue‐sarcoma resection was 3.1%, predominantly affecting liposarcoma‐patients. Post‐metastasis‐survival (PMS) was comparable for patients with AM and those with metastases elsewhere. Thus, patients at risk of AM may be followed‐up by regular abdominal‐sonography/CT‐scans.

## Data Availability

The data that support the findings of this study are available from the corresponding author upon reasonable request.
